# Management and Follow-up of Sporadic Cribriform-Morular Thyroid Carcinoma After the 2022 World Health Organization Reclassification

**DOI:** 10.1016/j.aed.2026.05.001

**Published:** 2026-05-12

**Authors:** Vanessa Cando-Bonilla, Ana Maria de Cunha Mercante, Rafael Loch Batista, Beatriz Godoi Cavalheiro, Ana O. Hoff, Luiz Paulo Kowalski, Marcos Tadashi Kakitani Toyoshima

**Affiliations:** 1 Department of Pathology, Hospital das Clínicas da Faculdade de Medicina da Universidade de São Paulo, São Paulo, Brazil; 2 Endocrinology Service, Hospital das Clínicas da Faculdade de Medicina da Universidade de São Paulo, São Paulo, Brazil; 3 Endocrine Oncology Service, Instituto do Câncer do Estado de São Paulo Octávio Frias de Oliveira, Hospital das Clínicas da Faculdade de Medicina da Universidade de São Paulo, São Paulo, Brazil; 4 Department of Head and Neck Surgery, Instituto do Câncer do Estado de São Paulo Octávio Frias de Oliveira, Hospital das Clínicas da Faculdade de Medicina da Universidade de São Paulo, São Paulo, Brazil; 5 Department of Head and Neck Surgery, Hospital das Clínicas da Faculdade de Medicina da Universidade de São Paulo, São Paulo, Brazil; 6 Laboratório de Carboidratos e Radioimunoensaio (LIM18), Hospital das Clinicas (HCFMUSP) Faculdade de Medicina, Universidade de São Paulo, São Paulo, Brazil

**Keywords:** cribriform-morular thyroid carcinoma, familial adenomatous polyposis, APC gene, colonoscopy, Wnt/β-catenin pathway

## Abstract

**Objective:**

Cribriform-morular thyroid carcinoma (CMTC) has been reclassified as a distinct thyroid tumor entity in the 2022 World Health Organization classification, no longer considered a subtype of papillary thyroid carcinoma. This redefinition raises important questions regarding postoperative management, including the need for radioactive iodine (RAI), thyroid-stimulating hormone (TSH) suppression, thyroglobulin monitoring, and screening for familial adenomatous polyposis (FAP). We report an apparently sporadic case and discuss a risk-adapted management approach.

**Case Report:**

A 24-year-old woman presented with a progressively enlarging right-sided neck mass. Thyroid ultrasound revealed a 2.8 cm predominantly solid, hypoechoic nodule (ACR TI-RADS 4), and fine-needle aspiration cytology was classified as Bethesda category V. Total thyroidectomy was performed. Histopathology confirmed CMTC with cribriform and morular architecture and focal vascular invasion, without extrathyroidal extension or nodal involvement (pT2*N*0). Immunohistochemistry demonstrated nuclear β-catenin expression and absence of thyroglobulin staining. There was no personal or family history of thyroid disease or polyposis. Colonoscopy was normal, and germline whole-exome sequencing did not identify pathogenic variants in adenomatous polyposis coli or other cancer-predisposition genes. Given the absence of high-risk features and no evidence of FAP, RAI therapy and TSH suppression were not administered. The patient remains disease-free after 24 months, under surveillance with periodic cervical ultrasonography.

**Discussion:**

CMTC management should not automatically follow conventional papillary thyroid carcinoma paradigms. In apparently sporadic, low-risk cases, a conservative postoperative strategy may be appropriate.

**Conclusion:**

This case supports an individualized, risk-adapted approach to CMTC, in which omission of RAI and aggressive TSH suppression may be reasonable without compromising oncologic safety.

**Highlights**

• Cribriform-morular thyroid carcinoma (CMTC) is no longer classified as a subtype of papillary thyroid carcinoma
• Exclusion of familial adenomatous polyposis (FAP) is essential after CMTC diagnosis
• Germline adenomatous polyposis coli testing helps rule out FAP in CMTC
• Thyroglobulin may be unreliable in CMTC surveillance
• A risk-adapted follow-up strategy may prevent overtreatment

**Clinical Relevance**

Cribriform-morular thyroid carcinoma was reclassified as a distinct entity in the 2022 WHO classification, creating uncertainty regarding postoperative management, particularly in sporadic cases. This report supports structured hereditary evaluation followed by individualized, risk-adapted follow-up once familial adenomatous polyposis has been reasonably excluded, which may help avoid unnecessary treatment and excessive surveillance.

## Introduction

Cribriform-morular thyroid carcinoma (CMTC) is a rare thyroid neoplasm, accounting for approximately 0.16% to 0.5% of all thyroid cancers^1^ and was reclassified as a distinct entity in the 2022 World Health Organization (WHO) classification of thyroid tumors.^2^ Previously considered a variant of papillary thyroid carcinoma (PTC), CMTC is now recognized as a biologically distinct tumor characterized by activation of the Wnt/β-catenin pathway and nuclear β-catenin expression.^1^, ^2^, ^3^

CMTC is frequently associated with familial adenomatous polyposis (FAP), typically driven by germline adenomatous polyposis coli (*APC*) mutations, and may represent the first clinical manifestation of this hereditary syndrome.^4^^,^^5^ Consequently, evaluation for FAP, including colonoscopic screening and germline *APC* testing when appropriate, is recommended after histopathological diagnosis.

However, the optimal postoperative management and surveillance strategy for apparently sporadic CMTC—after reasonable exclusion of FAP—remain uncertain. In the absence of dedicated guidelines, clinicians often extrapolate management from conventional PTC protocols, potentially leading to overtreatment. Here, we report a sporadic case of CMTC and discuss practical considerations for risk-adapted follow-up in the post-2022 WHO era.

## Case Presentation

A 24-year-old woman was referred for evaluation of a thyroid nodule after noticing a progressively enlarging right-sided cervical mass over approximately 5 months, occasionally associated with dysphagia. On physical examination, a palpable, fibroelastic, mobile thyroid nodule measuring approximately 3 cm was noted in the right thyroid lobe. Thyroid ultrasound revealed a 2.8 × 1.9 × 1.5 cm predominantly solid, hypoechoic nodule with regular margins located at the junction of the isthmus and the mid-to-lower portion of the right lobe, classified as ACR TI-RADS 4, without suspicious cervical lymphadenopathy. Fine-needle aspiration cytology was reported as Bethesda category V (suspicious for malignancy), demonstrating papillary and morular-like cell clusters with nuclear clearing and grooves and scant colloid. She subsequently underwent total thyroidectomy.

Surgical pathology revealed a unifocal CMTC located in the isthmus, measuring 2.8 cm in greatest dimension. The tumor was composed of well-demarcated tubular/cribriform structures with morular areas (Fig. 1 *A*). Mitotic activity was low (up to 1 mitosis per 2 mm^2^), and necrosis was absent. The lesion was nonencapsulated. Vascular invasion was identified in two foci, whereas no lymphatic or perineural invasion was observed. According to the 2025 American Thyroid Association criteria, this corresponds to focal vascular invasion (<4 foci). An illustrative example is provided in Supplementary Fig. 1. There was no microscopic or macroscopic extrathyroidal extension. Surgical margins were free of tumor, and no metastasis was identified in the single perithyroidal lymph node examined.

**Fig. 1:**
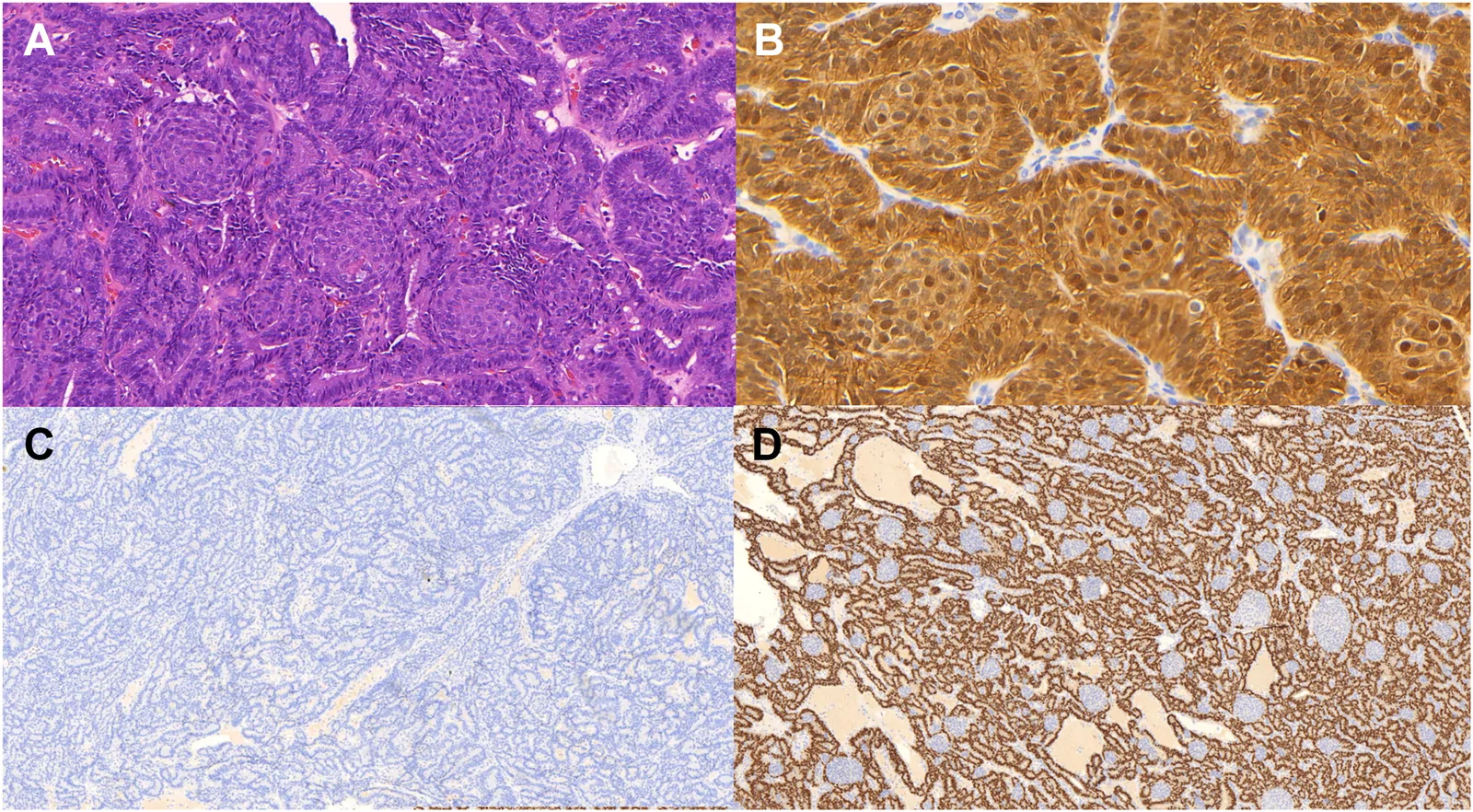
Histopathological and immunohistochemical features of cribriform-morular thyroid carcinoma. Legend: (A) Hematoxylin and eosin staining showing characteristic cribriform and morular architectural patterns (original magnification ×40). (B) Strong nuclear and cytoplasmic β-catenin immunoreactivity, consistent with activation of the Wnt/β-catenin pathway (×40). (C) Absence of thyroglobulin expression in tumor cells (×20). (D) Estrogen receptor positivity in the tubular and cribriform components (×20).

Immunohistochemistry demonstrated strong nuclear and cytoplasmic β-catenin expression (Fig. 1 *B*), absence of thyroglobulin expression (Fig. 1 *C*), preserved PAX-8 and TTF-1 expression, and low proliferative activity (Ki-67 < 5%). Estrogen (Fig. 1 *D*) and progesterone receptor expression were observed in tumor cells. These findings supported the diagnosis of CMTC in accordance with current WHO criteria.^2^

Preoperative thyroid function was within normal range (thyroid-stimulating hormone [TSH] 2.45 mIU/L, free T4 0.87 ng/dL). Following surgery, the patient was started on levothyroxine replacement (125 mcg/day; 2.0 mcg/kg/d), with subsequent dose adjustments aimed at maintaining TSH within the normal range rather than suppression. Postoperative laboratory evaluation showed transient hypocalcemia without persistent hypoparathyroidism.

Although thyroglobulin is considered an unreliable biomarker in CMTC, postoperative stimulated thyroglobulin was 8.4 ng/mL (TSH 55.1 mIU/L, anti-thyroglobulin antibodies <4 IU/mL). A nonstimulated thyroglobulin of 0.7 ng/mL was observed during follow-up.

There was no personal or family history of thyroid disease, thyroid cancer, or intestinal polyposis. Given the recognized association between CMTC and FAP, baseline evaluation for hereditary disease was undertaken. Colonoscopy showed no abnormalities. Postoperative whole-body radioiodine scan (^131^I) demonstrated low cervical uptake (48-hour uptake: 4.7%), with focal activity in the anterior cervical region consistent with residual thyroid tissue in the thyroglossal duct region and thyroid bed. No evidence of distant metastatic disease was identified.

Germline genetic assessment was performed using whole-exome sequencing (WES) on genomic DNA extracted from peripheral blood leukocytes. Variant analysis focused on rare coding and splice-site variants. No pathogenic variants or variants of uncertain significance were identified in *APC*, *CTNNB1*, *AXIN1*, or other cancer-predisposition genes.

In the absence of clinical, colonoscopic, or molecular evidence of FAP, the tumor was considered an apparently sporadic presentation of CMTC.

At the most recent follow-up (April 2026), approximately 24 months after surgery, the patient remains clinically and structurally disease-free. Postoperative evaluation included cervical ultrasonography and chest computed tomography, both of which showed no evidence of locoregional or distant disease. Surveillance has consisted of semiannual cervical ultrasonography. Given the frequent absence or unreliability of thyroglobulin expression in CMTC, postoperative monitoring has relied primarily on structural imaging rather than biochemical assessment. Thyroid hormone replacement was adjusted to maintain TSH within the normal range (TSH 2.98 mIU/L, free T4 1.04 ng/dL), with a levothyroxine dose of 100 mcg/day (1.6 mcg/kg/d).

## Ethics Statement

This study was approved by the local Institutional Review Board. Written informed consent was obtained from the patient for publication of this case and accompanying images.

## Discussion

CMTC has historically been linked to FAP due to its association with germline *APC* mutations and Wnt/β-catenin pathway activation, shaping routine genetic and colorectal evaluation at diagnosis.^1^

### CMTC Beyond Syndromic Disease

Although CMTC is a recognized extracolonic manifestation of FAP, a substantial proportion of cases arise sporadically. These tumors may be histologically indistinguishable from syndromic CMTC but differ in molecular context, with somatic Wnt/β-catenin pathway alterations reported in sporadic cases.^1^^,^^4^, ^5^, ^6^, ^7^ While CMTC can precede the diagnosis of FAP,^8^ the prevalence of germline APC mutations in unselected cohorts appears relatively low. In this context, the optimal strategy for long-term colorectal surveillance remains uncertain.

CMTC predominantly affects young women, with a marked female predominance. Although estrogen and progesterone receptor expression has been consistently described in tumor cells, its clinical significance remains unclear. It has been hypothesized that hormonal signaling may contribute to tumor biology or partly explain the striking sex distribution; however, there is currently no evidence supporting a role for hormonal status in prognosis or therapeutic decision-making.^1^^,^^3^^,^^4^

The 2022 WHO reclassification of CMTC as a distinct entity further underscores its biological heterogeneity but provides no specific guidance regarding genetic evaluation or long-term surveillance.^1^^,^^2^^,^^5^

### Reassessing the Role of Colorectal Screening

Baseline colonoscopic evaluation remains appropriate after a diagnosis of CMTC to exclude unrecognized FAP,^9^ particularly as genetic testing may not be readily available in all clinical settings and because thyroid carcinoma may precede clinically detectable colonic abnormalities in approximately 50% of patients with FAP.^4^^,^^10^ In our case, germline WES was obtained after the initial evaluation and did not identify pathogenic variants in *APC*. According to the *NCCN Genetic/Familial High-Risk Assessment: Colorectal guidelines*, individuals who test negative for pathogenic *APC* variants and have fewer than 10 adenomas should follow average-risk colorectal cancer screening recommendations, including colonoscopy every 10 years for those aged 45–75 years.^11^

However, data specific to CMTC remain limited, and no evidence-based surveillance recommendations can currently be established. An individualized approach integrating age, family history, and molecular findings may better balance cancer prevention with avoidance of unnecessary interventions.

### Genetic Testing: Targeted versus Broad Approaches

CMTC differs molecularly from conventional PTC, being primarily driven by Wnt/β-catenin pathway alterations rather than typical MAPK pathway mutations.^12^ CMTC exhibits a distinctive immunophenotype, including strong nuclear and cytoplasmic β-catenin expression and frequent absence of thyroglobulin staining, which underscores its biological distinction from conventional follicular-derived differentiated thyroid carcinoma.^1^

Genetic evaluation therefore plays a central role in distinguishing syndromic from apparently sporadic disease. Germline multigene panel testing, including *APC*, is the recommended strategy for diagnosing FAP.^11^ In settings where access to validated multigene panels is limited, broader approaches such as WES may serve as an alternative, although with potentially lower analytical sensitivity for specific genes compared with targeted panels.^13^ In the present case, germline WES was performed on peripheral blood and did not identify pathogenic variants or variants of uncertain significance in *APC*, or other polyposis-associated genes, including *CTNNB1* and *AXIN1*—key components of the Wnt/β-catenin pathway—supporting an apparently sporadic presentation. However, as sequencing was limited to germline DNA, the somatic molecular driver of the tumor could not be characterized. This is particularly relevant, as sporadic CMTC is typically driven by somatic alterations in the Wnt/β-catenin pathway.^1^ While a negative exome does not fully exclude rare mechanisms such as mosaicism or noncoding variants, when integrated with a negative family history and normal colonoscopy, it markedly reduces the likelihood of an underlying germline syndrome.

## Implications for Follow-up After the WHO 2022 Reclassification

The reclassification of CMTC as a distinct entity raises important questions regarding the appropriateness of applying conventional differentiated thyroid carcinoma follow-up strategies, including routine radioactive iodine therapy, long-term TSH suppression, and thyroglobulin-based surveillance.^1^^,^^14^

Vascular invasion is classified as an intermediate-risk feature in the 2025 American Thyroid Association risk stratification system for differentiated thyroid carcinoma,^10^ and was present in our patient. However, the clinical significance of vascular invasion in CMTC remains uncertain, particularly when limited in extent. In our case, vascular invasion was focal and occurred in the absence of other high-risk features, such as extrathyroidal extension, nodal involvement, or high proliferative activity. This distinction is clinically relevant, as focal vascular invasion may not carry the same prognostic implications as extensive vascular invasion described in conventional differentiated thyroid carcinoma. Importantly, CMTC differs biologically from conventional PTC, particularly due to its frequent lack of thyroglobulin expression and uncertain responsiveness to radioactive iodine therapy. These differences challenge the direct application of standard risk stratification models derived from PTC. Angioinvasion and capsular invasion have been described in approximately 30% and 40% of CMTC cases, respectively. Lymph node metastases occur in about 10% to 12% of patients, and distant metastases in roughly 5%, while aggressive clinical behavior is predominantly observed in rare high-grade carcinoma.^1^^,^^3^^,^^15^^,^^16^ In one of the largest single-center series with long-term follow-up, no recurrences or disease-specific deaths were observed over a mean follow-up of 15 years, even among cases with vascular invasion.^17^ These findings suggest that the prognostic significance of vascular invasion in CMTC may differ from that observed in conventional papillary or follicular thyroid carcinoma.

The present case also exhibited a nonencapsulated growth pattern, which, together with vascular invasion, deviates from the typically well-demarcated presentation of CMTC.^18^ In differentiated thyroid carcinoma, these features are generally associated with a higher risk of adverse outcomes. Although their prognostic significance in CMTC remains uncertain, they may indicate a subset of tumors with higher-risk features.

Only one perithyroidal lymph node was examined in this case, reflecting a limited nodal assessment. However, prophylactic central neck dissection was not performed, as there was no preoperative or intraoperative evidence of nodal disease. The excised lymph node was removed incidentally during thyroidectomy. While this approach is consistent with current surgical practice in clinically node-negative patients, it represents a limitation in accurately assessing nodal status and may result in understaging.

These data indicate that CMTC is not uniformly indolent; however, most reported cases—particularly sporadic, unifocal tumors without extrathyroidal extension—appear to follow a relatively favorable clinical course after adequate surgical treatment.^1^ Current data indicate that hemithyroidectomy may be sufficient for apparently sporadic, unifocal CMTC lacking high-risk features, whereas total thyroidectomy is typically recommended in FAP-associated disease due to a higher likelihood of multifocality.^1^^,^^5^ In our case, total thyroidectomy was performed based on preoperative suspicion of malignancy (Bethesda V cytology) and clinical decision-making prior to definitive histopathological diagnosis. The tumor location in the isthmus and its size (2.8 cm) were also considered relevant factors supporting the surgical approach. Notably, studies in PTC have shown that isthmic tumors may be associated with higher rates of lymph node metastasis and more aggressive features, which often favor total thyroidectomy.^19^^,^^20^ However, there are no specific data regarding the prognostic significance of isthmus location in CMTC. In retrospect, a more conservative surgical approach, such as lobectomy or isthmusectomy, could be considered in selected low-risk cases of CMTC.

Radioiodine therapy was not administered in this case. This decision was based on the distinctive molecular phenotype of CMTC, characterized by constitutive activation of the Wnt/β-catenin pathway, which is associated with impaired follicular differentiation and reduced expression of key proteins involved in iodine handling, including the sodium-iodide symporter, thyroperoxidase, and thyroglobulin. As a result, the likelihood of clinically meaningful radioiodine uptake is considered low. Unlike BRAF-driven PTCs, CMTC belongs to the non-BRAF-non-RAS molecular subtype, and its iodine-refractory phenotype is not amenable to MAPK-targeted redifferentiation strategies.^18^ Furthermore, available evidence suggests that CMTC generally follows a favorable clinical course after surgery alone, with low rates of recurrence and disease-specific mortality.^4^ In addition, data from low-risk differentiated thyroid carcinoma indicate no clear benefit of postoperative radioiodine therapy in similar clinical contexts.^21^

In our patient, the absence of structural disease on postoperative imaging, together with focal vascular invasion and no other high-risk features, supported a conservative management strategy without radioiodine therapy.

A diagnostic whole-body radioiodine scan was performed and demonstrated focal cervical uptake consistent with residual thyroid tissue in the thyroglossal duct region and thyroid bed, without evidence of iodine-avid structural disease. Given the biological features of CMTC, the clinical utility of diagnostic whole-body scanning in this setting may be limited, and its omission could be considered in selected patients with similar clinicopathological profiles.

The genomic landscape of CMTC—characterized predominantly by Wnt/β-catenin pathway activation and relative absence of typical MAPK pathway drivers—further challenges the automatic application of management paradigms developed for conventional PTC.^12^ These findings highlight that CMTC should not be managed as a direct surrogate of conventional PTC, and that extrapolation of risk stratification models may lead to overtreatment.

In patients without clinical or molecular evidence of FAP, a conservative and structure-based follow-up strategy may therefore be appropriate, rather than intensified surveillance driven by presumed hereditary risk.

In advanced or refractory cases, systemic therapies may be considered. However, such scenarios are uncommon in apparently sporadic CMTC, which typically follows a favorable clinical course after surgery alone. Given the molecular profile of CMTC, conventional radioiodine-based approaches are often ineffective. Targeted therapies, including multikinase inhibitors, have been proposed based on experience with other thyroid carcinomas, although evidence in CMTC remains limited. In addition, the frequent expression of estrogen and progesterone receptors has raised interest in the potential role of antiestrogen therapies; however, their clinical efficacy has not been established.^1^

In practical terms, CMTC should prompt structured initial evaluation for hereditary disease. Once a germline syndrome has been reasonably excluded, management intensity should be guided by individualized oncologic risk rather than historical assumptions. A simplified, risk-adapted follow-up strategy centered on clinical assessment and periodic neck ultrasonography may help avoid overtreatment while maintaining appropriate surveillance in the post–WHO 2022 era (Fig. 2). Although TSH suppression is not routinely indicated in low-risk CMTC, it may be considered in selected high-risk cases. This approach is extrapolated from differentiated thyroid carcinoma, where TSH suppression is associated with reduced recurrence risk. However, its benefit in CMTC remains uncertain, and decisions should be individualized based on disease extent and overall risk profile.

**Fig. 2:**
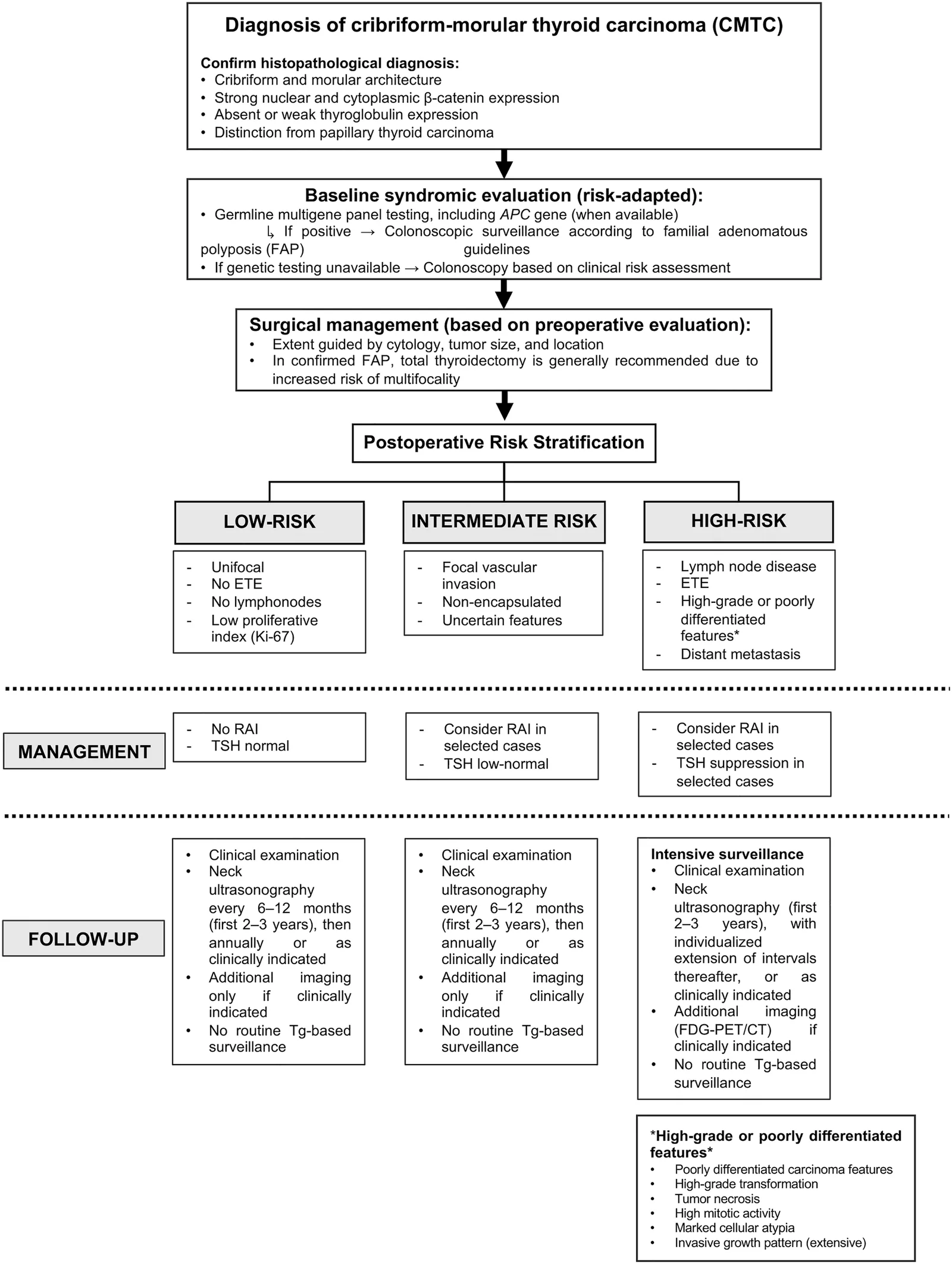
Proposed diagnostic and management algorithm for cribriform-morular thyroid carcinoma in the post-2022 World Health Organization classification era, Legend: A clinical decision pathway for cribriform-morular thyroid carcinoma (CMTC). Following histopathological confirmation, a structured, risk-adapted baseline evaluation for hereditary disease is recommended, comprising germline multigene panel testing (such as *APC*) when available, along with colonoscopic assessment according to clinical context. Initial surgical management is guided by preoperative evaluation, while postoperative management is tailored according to risk stratification. In low-risk cases, routine radioactive iodine (RAI) therapy and TSH suppression are not indicated, and surveillance is primarily based on clinical assessment and periodic neck ultrasonography. In intermediate- and high-risk settings, management may be individualized, including consideration of RAI and TSH suppression in selected cases. Given the absence of CMTC-specific guidelines, risk stratification and management recommendations—particularly for intermediate- and high-risk categories—are informed by extrapolation from differentiated thyroid carcinoma. Surveillance intervals, TSH targets, and the preoperative basis of surgical decision-making are indicated within the algorithm. TSH, thyroid-stimulating hormone

### Limitations

This report has limitations inherent to single-case observations. The follow-up duration remains relatively short, and longer surveillance is required to confirm the durability of this favorable course. Additionally, nodal assessment was limited, as only one perithyroidal lymph node was examined. No prophylactic central neck dissection was performed, given the absence of preoperative or intraoperative evidence of nodal disease, and the lymph node was removed incidentally during thyroidectomy; therefore, the possibility of understaging cannot be excluded.

Molecular testing for somatic alterations (including *BRAF*, *TERT* promoter, *RAS* mutations) was not performed in this case. Although not required for clinical decision-making, such analyses could provide further insight into the molecular mechanisms underlying tumor development, particularly in the context of Wnt/β-catenin pathway activation, which is characteristic of CMTC.

Furthermore, although comprehensive germline analysis was negative, rare mechanisms such as mosaicism or noncoding variants cannot be completely excluded. Larger multicenter studies are needed to better define evidence-based surveillance strategies in CMTC.

## Conclusion

CMTC should not be regarded as an automatic surrogate marker for hereditary cancer syndromes nor managed reflexively according to conventional differentiated thyroid carcinoma paradigms. In the post–WHO 2022 era, histological diagnosis alone should not mandate uniform long-term syndromic surveillance or routine application of radioiodine therapy and thyroglobulin-based monitoring once comprehensive initial evaluation has reasonably excluded FAP.

Rather than diminishing the importance of appropriate genetic and endoscopic assessment, management should integrate clinical context, family history, tumor characteristics, and molecular findings to align surveillance intensity with true oncologic risk.

## Data Availability Statement

The data supporting the findings of this study are available from the corresponding author upon reasonable request. Due to patient privacy considerations, the data are not publicly available.

## Disclosure

The authors have no conflicts of interest to disclose.
