# Supplementary material for: Management and Follow-up of Sporadic Cribriform-Morular Thyroid Carcinoma After the 2022 World Health Organization Reclassification
Source: AACE Endocrinol Diabetes. 2026 May 12;13(5):679–84. doi: 10.1016/j.aed.2026.05.001 (PMC13728255; doi:10.1016/j.aed.2026.05.001)
Supplement: Supplementary file 1 [file mmc1.docx]

**Management and Follow-up of Sporadic Cribriform-Morular Thyroid Carcinoma After the 2022 WHO Reclassification**

**Supplementary Figure S1.** Microphotograph demonstrating vascular invasion. Immunohistochemical staining for CD31 highlights endothelial-lined vascular channels containing intraluminal tumor cell clusters, confirming true vascular invasion (original magnification ×20).
